# Egyptian Fellahs

**Published:** 1876-06

**Authors:** 


### EGYPTIAN FELLAHS.

The philosopher who deemed him happiest who has the fewest wants ought to have been an Egyptian fellah. He is sometimes even born in the fields. The women work up to the day of their confinement. They lie up one day and are out again the next, and the baby is laid near them in the fields on a bit of sacking. Ignorance and poverty lead to other sad consequences. Premature old age comes on at forty, and the population is kept down by a terrible infant mortality. Out of the 140,000 annual deaths, 80,000 are of infant children. It has been calculated that three out of every five that are born die before the age of two. For those that survive, an old Egyptian custom that is still practiced is symbolical of their future. The child is put into a sieve and rolled about to the beating of drums. "It is in order to harden him," say the people.

